# World Health Organization Evidence-Based Self-Help Plus Intervention for Stress Management via Chatbot: Protocol for Adaptation to a Tech-Enabled Model

**DOI:** 10.2196/69644

**Published:** 2025-06-26

**Authors:** Valentina Fietta, Silvia Rizzi, Lorenzo Gios, Maria Chiara Pavesi, Chiara De Luca, Silvia Gabrielli, Merylin Monaro, Nicolò Navarin, Erik Gadotti, Oscar Mayora-Ibarra, Marianna Purgato, Corrado Barbui, Stefano Forti

**Affiliations:** 1 Department of General Psychology University of Padua Padua Italy; 2 Digital Health Research, Centre for Digital Health & Wellbeing Fondazione Bruno Kessler Trento Italy; 3 TrentinoSalute4.0 - Competence Center for Digital Health, Province of Trento Fondazione Bruno Kessler Trento Italy; 4 Istituto Pavoniano Artigianelli Trento Italy; 5 Department of Mathematics University of Padua Padua Italy; 6 WHO Collaborating Centre for Research and Training in Mental Health and Service Evaluation, Department of Neurosciences, Biomedicine and Movement Sciences, Section of Psychiatry University of Verona Verona Italy

**Keywords:** digital health, virtual coach, mobile application, breast cancer, pregnancy, stress management

## Abstract

**Background:**

This paper describes the adaptation of an evidence-based intervention for mental health, Self-Help Plus (SH+), to a digital platform, aiming to expand mental health support through innovative technological solutions. The SH+ intervention, designed by the World Health Organization (WHO), is a low-intensity, self-guided program aimed at improving mental well-being. The intervention has been integrated into a chatbot-driven mobile app called “ALBA” (Automated Well-Being Assistant).

**Objective:**

This protocol describes the stages of transition (porting) from a traditional face-to-face, group-based approach to a tech-enabled model, tailored specifically for women with breast cancer and pregnant women. The adaptation of this approach is described, focusing on 2 key aspects: (1) the shift from in-person delivery to a tech-enabled intervention and (2) the customization of the intervention to address the unique needs of the selected target groups.

**Methods:**

The development of the ALBA app involved a collaborative, multidisciplinary approach combining expertise in psychology, eHealth IT, interaction design, and specific domain knowledge related to pregnancy and oncology. The development process followed 2 primary methodologies: user-centered design and service design. These approaches emphasize understanding user needs, promoting iterative improvements, and continuously incorporating user feedback. The development followed the Obesity-Related Behavioral Intervention Trials (ORBIT) model, which involves a cycle of literature review, stakeholder consultation, content development, software development, and evaluation. At the same time, specific attention was dedicated to (1) adapting the SH+ protocol to suit the chatbot-driven ALBA platform, (2) tailoring content to the target population, and (3) incorporating interactive features to improve engagement and potential efficacy.

**Results:**

The manuscript outlines the key steps and processes involved in adapting the SH+ intervention into the ALBA digital platform. This includes an overview of the challenges and opportunities of translating a face-to-face, group-based intervention into a tech-enabled model and provides insight into how customization for specific populations, such as women with breast cancer and pregnant women, was integrated into the design of the platform. This is in view of informing future studies on the development and adaptation of already validated protocols into mobile health (mHealth) interventions in the field of mental health and mental well-being.

**Conclusions:**

The digital adaptation of the SH+ intervention into the ALBA platform could represent a significant step forward in expanding the accessibility and personalization of mental health interventions in potentially different settings and target groups. The use of virtual coaching applications can play a central role in improving the availability and ease of access of psychological support, even in different and mHealth formats. Future developments could include further testing in real-world settings to expand (1) adaptations for various target groups and (2) the purpose of use to range from primary prevention to more clinically focused interventions.

**International Registered Report Identifier (IRRID):**

DERR1-10.2196/69644

## Introduction

### Theoretical Framework

The current scientific literature shows an increasing demand for psychological support within the general population, with even higher levels when considering vulnerable populations exposed to stressful situations [1]. The World Health Organization (WHO) strategy for global mental health is based on principles of inclusivity and scalability, covering the entire arch of mental health promotion and prevention for citizens exposed to the risk of mental distress. The gap between the prevalence of mental health problems and the services available to those in need of specific interventions represents a current public health challenge, and WHO has further underlined this issue through the “mental health Global Action Program” [2], where particular attention is also paid to the potential role of digital technologies in facilitating both availability of services and optimized delivery of the intervention [3]. Digital health is considered a pivotal strategy to promote equitable, affordable, and universal access, reducing the gap between services and those who can benefit from receiving mental health interventions [4]. Indeed, an increasing body of literature confirms the potential of new technologies—including telemedicine, mobile health (mHealth) initiatives, and digital therapeutics (DTx)—for promoting a continuum of care from clinic to home and a stepped-care approach. In particular, low-intensity mental health interventions could be considered appropriate for implementation within digital health solutions [5]. They usually refer to specific programs where the active involvement of health care staff and specialists is not necessarily required. They are based on solid principles from evidence-based psychological interventions embedded into a self-help approach. Among them, WHO is promoting Self-Help Plus (SH+), a low-intensity intervention for stress management initially developed for hard-to-reach vulnerable population groups [6]. These types of interventions are usually designed to be transdiagnostic and easily adaptable in different contexts. Because of their general structure and purpose, low-intensity approaches can be easily used to increase access to viable and evidence-based psychological interventions to a broader target audience, reporting either limited or mild symptoms linked with mental distress or illness within a stepped-care approach [7]. The potential combination of evidence-based, low-intensity interventions and mHealth approaches could lead to an effective, sustainable, and inclusive strategy for improving the scalability of mental health interventions both in terms of prevention and treatment [8].

### Intervention Characteristics

#### Self-Help Plus (SH+)

SH+ is a transdiagnostic intervention based on acceptance and commitment therapy, a third-wave cognitive behavior therapy (CBT) [9]. SH+ is structured into 5 sessions comprising audio recordings featuring mindfulness practices and exercises and aiding participants with recognizing barriers and facilitators related to stress management. Several studies report positive effects of SH+, considering long-term efficacy in a target population of refugees and asylum seekers [10-15]. Other studies show a still-debatable effect of SH+ when used with health care professionals [16].

SH+ is particularly suitable for a digital version, considering that (1) it is largely based on third-wave CBT techniques, which are usually more suitable than other psychotherapeutic approaches for transferring into interventions delivered by mobile apps [9], and (2) it includes self-help materials, like the illustrated Doing What Matters in times of stress (DWM) booklet [17] encompassing exercises designed to reduce stress and increase social support networks, adaptive coping mechanisms, and resilience. The latter has recently been converted into a web app for populations in war (RESPOND PROJECT) [18].

#### Target Populations: Public Health Issues and Sustainability

The adaptation model is designed to target two specific groups usually exposed to stress and potentially stigmatized when accessing mental health services: women with breast cancer and pregnant women.

Physical illnesses like breast cancer often coexist with psychological distress, including stress, anxiety, and depression [19]. Furthermore, chronic fatigue, sleep problems, body image disturbance, and diminished social interactions emerge as characteristic comorbidities. Additionally, women diagnosed with primary breast cancer continue to exhibit susceptibility to psychological ailments over an extended duration, underscoring the profound influence of this medical condition on patients’ quality of life [19]. It has been observed that a positive psychological attitude improves disease management and recovery, underscoring the significance of addressing mental health issues in addition to physical ones [20]. Numerous studies have highlighted the efficacy of CBT interventions for improving mental health and overall quality of life for women with breast cancer [21,22].

A second target group for this exercise is pregnant women. The transition to motherhood is an event that entails a significant change in a woman’s life. Pregnancy is characterized by major transformations in the woman, with an impact on her physical, mental, and social well-being. The literature shows that pregnant women experience various psychological symptoms, the most frequent being anxiety, stress, and depression [23]. Thus, women in the perinatal period may experience worries and the fear of being unable to manage the changes associated with the period and the baby or children yet to be born [24]. To date, psychoeducational interventions that promote women’s psychological well-being during pregnancy are scarce and tend to focus mainly on samples of women with psychiatric symptoms (eg, perinatal depression disorder) [25].

Despite the demonstrated effectiveness of psychological support interventions at alleviating stress in these target populations, only a negligible proportion of women belonging to these groups have access to psychological support. Indeed, the need for public resources and mental health care services is increasing due to the aging population and the rising prevalence of cancer and diseases that accompany mental illness. However, there are several challenges to accessing mental care services. For example, only 17% and 28%, respectively, of Italian and German women with breast cancer benefit from psycho-oncological support [26,27]. On the other hand, in a Portuguese study, only 13.6% to 20.2% of women screened positive for possible depressive disorders sought help during pregnancy [28].

Many women face geographical barriers, with specialized centers often located far away and limited availability of qualified personnel. Stigma and self-stigma can also prevent patients from seeking psychological support [29]. 

#### Opportunities From New Technologies

The use of digital tools has been explored as a means of providing psychological support in the midst of mental health care accessibility and sustainability challenges. DTx has surfaced as a convenient and readily accessible alternative for individuals pursuing psychological support [30]. They offer evidence-based and clinically evaluated digital mental health interventions delivered through software, addressing various diseases and disorders by providing treatment, management, and preventive measures [31]. mHealth services could improve accessibility and standardization of service delivery, as well as reduce costs and potential stigma associated with access to service through a stepped-care approach enabled by technologies [32]. mHealth and DTx might support a patient-centric approach, empowerment, and self-care despite the potential lack of human contact [33]. Using digital tools to promote psychological well-being can be an optimal solution specifically for pregnant women and those with breast cancer, considering the flexibility and adaptability to patients’ needs characterizing these tools, as well as reduced social stigma [34]. The literature reports promising results concerning the effectiveness of DTx based on CBT targeting psychological well-being for both our target groups [35-38].

### Objectives

The primary aims of this paper are as follows:

1. To map a solid pathway to design and adapt an SH+ protocol into a technology-enabled intervention, for implementation through a novel technological tool, the chatbot ALBA (Automated Well-Being Assistant).
2. To emphasize the specific adaptations of a digitally delivered, low-intensity intervention when used with subpopulations particularly exposed to social-psychological stressors, such as women with breast cancer and pregnant women.

## Methods

This section reports the development of a virtual coaching solution named ALBA. Subsequently, the section clarifies the adaptation process of the SH+ protocol into a mobile app.

### Process Overview

The development of the intervention leveraged the integration of different expertise, including psychology, eHealth IT, interaction design, and domain knowledge on pregnancy and oncology. A multidisciplinary team was created to account for the users’ different needs and to accommodate for psychological consistency of the intervention and the proper adoption of IT requirements and user-design principles in the process, to ensure high quality in terms of adaptability of the proposed tools in the digital context.

**Figure 1:**
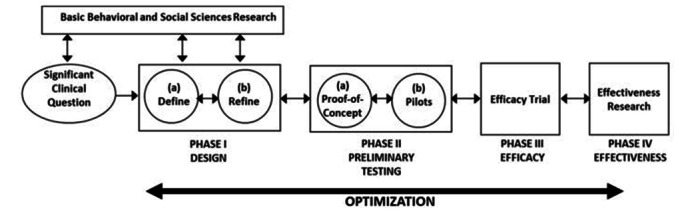
The Obesity-Related Behavioral Intervention Trials (ORBIT) model [41].

The entire development of the intervention is based on two main methodologies: user-centered [39] and service design [40]. These approaches emphasize understanding and meeting user needs, promoting iteration and continuous improvement based on user feedback. SH+ will be developed iteratively, following the Obesity-Related Behavioral Intervention Trials (ORBIT) model [41], as illustrated in Figure 1. The development process included a literature review; exploration, incorporating input from user representatives, health care providers, and eHealth experts, including designers and developers; intervention content development, which was identified and adjusted from the SH+ intervention manual; iterative software development and formative evaluation; and finally, considerations regarding privacy, security, and organizational anchoring.

After the preliminary phase of literature review and integration of the WHO protocol, we systematically carried out the subsequent steps to develop the ALBA chatbot prototype. Following the ORBIT development process ensures a scientifically step-by-step approach to recognize standardized creation of digital behavior change interventions. We then progressed to the initial stage of the ORBIT model, specifically Phase 1a (Define). This phase is designed to establish a solid foundation for the intervention by clearly defining the clinical problem to be addressed as well as the targets and ensuring that the intervention is conceptually sound and grounded in relevant theory and empirical evidence. In line with this, our goal was to adapt and refine the core components of SH+ prior to feasibility testing. Accordingly, this paper focuses on the initial adaptation methodology, and the process is described in detail in the following sections and summarized in Table S1 (see Multimedia Appendix 1). In contrast, real-world usage metrics, such as dropout rates, session completion, and participant feedback, will be addressed in later phases, including preliminary testing (Proof of Concept and Pilots) first and subsequent efficacy and effectiveness trials. These results will be presented in future publications.

### Adaptation from SH+

#### Content

The SH+ package has the following 3 main components: a prerecorded audio course, a facilitator manual, and the DWM booklet. All materials have been downloaded and translated (if necessary) from the WHO website [42]. The audio material imparts key information about stress management and guides participants through individual exercises and small group discussions. The innovation is that main intervention components are delivered as intended through the use of prerecorded audio, without the burden of extensive training and supervision to target hard‐to‐reach populations. SH+ is structured into 5 sessions based on acceptance and commitment therapy for stress management, namely (1) grounding, (2) unhooking, (3) acting on your values, (4) being kind, and (5) making room. The initial phase involved carefully examining the guide manual and audio. A judicious assimilation of selected components from the DWM booklet complemented this endeavor. Emphasis was placed on augmenting the visual and illustrative facets, notably by incorporating cartoon- and comic book–like elements.

**Figure 2:**
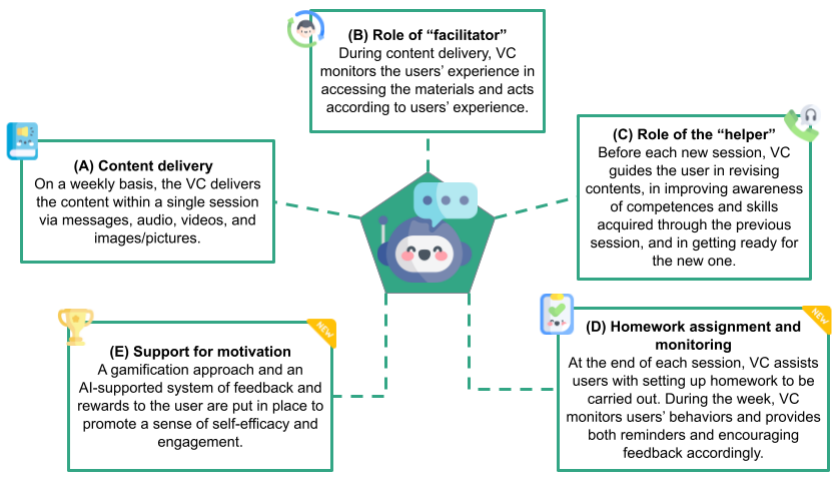
Automated Well-Being Assistant (ALBA) chatbot and its functionalities as a virtual coach (VC). AI: artificial intelligence.

The working group then ventured into adapting the group and in-person intervention to an individually delivered approach through a chatbot on an app. This transposition required various human and logistical factors to be changed or reconsidered to accommodate the new mode of psychological support delivery. ALBA is responsible for disseminating diverse content forms such as textual materials, auditory cues, video presentations, and graphical depictions (Figure 2A). The auditory components about SH+ exercises, alongside visual elements, are slated for comprehensive modification, tailored to suit an intervention delivered through mobile devices. Furthermore, the integration of psychoeducational elucidations of complex concepts is realized through videos or graphical representations. This strategic approach was conceived in response to the challenges posed by the transfer of substantial didactic information within the confines of a chatbot-mediated interaction.

#### Guide

The facilitator guide manual of SH+ helps trained nonspecialist facilitators conduct the course using prerecorded audio materials. The facilitators run the group, run discussions, and answer any questions. They also make sure the group runs safely. They have a limited role in explaining content as this mostly comes from the audio. In the RESPOND interventions [18], the user also receives weekly support through a 15-minute telephone call from a “helper.” The helper, as a trained individual, adheres to a predefined script and is responsible for initiating weekly telephonic interactions with the participant. These interactions serve multiple purposes of supporting, encouraging, problem-solving, and vigilantly tracking the participants’ advancements along the trajectory of stress management.

The conventional role assumed by facilitators in the physical realm is supplanted within this context by ALBA. This automated agent guides participants throughout the sessions (Figure 2B). ALBA uses prewritten scripts with the option to provide personalized responses based on user button choices and free text answers. Although this approach may not offer the same level of customization as generative methods, it ensures SH+ protocol transposition standardization and safeguards against potential issues such as hallucinations and biases commonly associated with generative artificial intelligence models [43]. The intervention was constructed in such a way that ALBA is always the first to interact with the user. It is currently not possible for the user to ask specific questions. Additionally, the chatbot functions in an interactive capacity, prompting moments of introspection and contemplation. It engages participants through a series of inquiries, encompassing both directed and open-ended questions, thereby soliciting feedback pertaining to the comprehensibility and lucidity of the disseminated subject matter. ALBA will also assume a role similar to that of the helper within the RESPOND protocol framework (Figure 2C). Its operational function encompasses the supervision and oversight of exercise implementation, primarily bolstering participant engagement and fostering intervention adherence. Notably, integrating these roles constitutes an innovative and resource-efficient approach to orchestrating the participant’s progression through the sessions. Moreover, this amalgamation inspires the participant with a consistent and reassuring sense of guidance.

### Adaptation for the Target Populations

#### Participants

Participant recruitment will be conducted through word of mouth and direct acquaintances and by advertising the study within public health care settings. Only volunteers aged ≥18 years will be included. All data will be collected confidentially with participants’ informed consent. For more details about co-design participant recruitment, see our previous study [44].

A thorough evaluation of the psychological states of prospective female participants is undertaken before they engage in the intervention. This evaluative process encompasses an appraisal of key indicators of mental well-being, thereby facilitating the discernment of suitability for study inclusion. This procedural component serves as a crucial gatekeeping mechanism and furnishes a foundational benchmark against which the eventual intervention’s effectiveness can be gauged. Implementing baseline and subsequent temporal administration of questionnaires yields a comprehensive and scientifically based view, thereby illuminating the trajectory and impact of the final intervention over time. Regarding materials, they have been translated into Italian and culturally adapted for the target populations. Exemplary content designed for humanitarian emergencies is redrafted to suit the context of stressful events experienced by Italian women (eg, typical familiar environment, medical conditions). The chatbot’s functionality is attuned to the distinctive requirements of the designated populations. To elucidate, all pertinent materials are tailored to encompass a prototype generic Italian female user, incorporating requisite specific adaptations (eg, Italian feminine adjectives and word desinences). Throughout the whole design process, a careful assessment is directed toward the potential transmutation of exercises into a stress-inducing factor (eg, focusing on body exercises for women diagnosed with breast cancer).

#### Session

**Figure 3:**
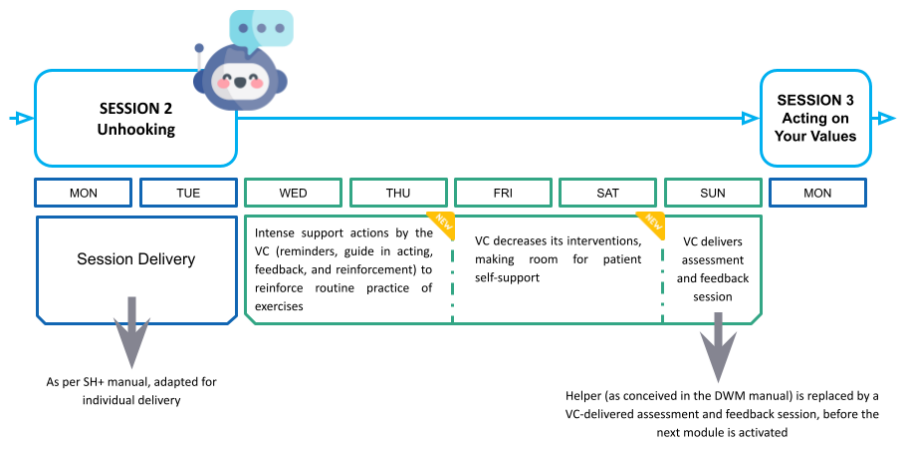
Representation of the intervention’s structure in an exemplary week (Week 2: Session Unhooking). DWM: Doing What Matters in times of stress; SH+: Self-Help Plus; VC: virtual coach.

The original SH+ intervention is organized in 2‐hour sessions and groups of 20 to 30 people on an established weekly schedule. ALBA releases dialogue sessions in this digital translation, but the intervention is literally in the end user’s hands, offering a self-help intervention wherever she wants. Moreover, the user can personalize the session’s day. The dialogue duration is fixed at 40 minutes. To support adherence, the session can be divided into 2 mini sessions on 2 subsequent days, preventing user fatigue and improving the usability of both the chatbot and the app. After completing a session, participants receive supportive and motivational reminders and feedback (Figure 3). This facilitates the establishment of a routine, increasing exercise completion in populations with different personal and time management needs, like pregnant women and women with breast cancer.

### Key Points From Chatbot and mHealth Literature

#### Homework

User interface design and feedback are essential for personalized behavior change support systems. Incentives and reminders are useful strategies that facilitate adherence, learning, and safety during self-guided interventions [45,46].

ALBA will assign homework during the session delivery. This homework consists of exercises that should be carried out by the users before the following session, as per the facilitator’s instructions. The diary section will support self-monitoring of exercise progress and completeness of sessions (Figure 2D). It is within this section that all reminder and feedback controls are created. Indeed, the novelty of this app is that the completion of exercises is monitored in the evenings before the next session, reinforcing engagement, which is different from standard SH+ in-person delivery. This will be achieved through the strategic incorporation of gamification elements (like badges) and timely feedback prompts interposed between sessions. This paper will not discuss all these sets of sophisticated IT rules and components.

#### Engagement

The design and deployment of conversational agents, such as chatbots, as virtual coaching solutions allow for maintaining the intuitiveness and naturalness of dialogue-based interaction while exploiting the benefits of full automation [47,48]. Moreover, gamification in health care is a promising way to improve patient adherence. It can make interventions more fun and engaging, increase patient awareness, and give patients ownership over their treatment [49].

Our app has a main page where users can dialogue with ALBA by selecting the toolbar button. It also includes other sections, as follows: the “Gallery,” featuring educational videos and intervention introductions; the “Diary,” strictly connected with the “Exercises” page with all available self-help exercises; and the “Progress” section providing users with an overview of their “journey” toward increased well-being. Here, users can see the sticker badges earned by practicing exercises between sessions appearing on their suitcase illustration, serving as long-term positive rewards. Badges also have symbolic meanings for psychological instruments and techniques acquired during the intervention. Instead of human helpers, the avatar-like chatbot serves as the primary support system, giving feedback and rewards, promoting a sense of accomplishment, and facilitating user motivation (Figure 2E).

### Ethical Considerations

All future studies’ data and our previous preliminary data [44] are confidentially collected in Italian and deidentified with participants’ informed consent. Eventually, confidential audio recordings of semistructured interviews are used for data analysis, and participants are identified only by numeric codes. At the studies’ conclusion, participants can request the research outcomes from the research manager. Participants did not receive any compensation. A local ethics committee will approve all future studies, as with our previous co-designed study [44]. Privacy issues require strict adherence to informed consent protocols and secure data retention compliant with the General Data Protection Regulations. Finally, the participants will be informed of the study’s results through the dissemination of articles.

## Results

**Figure 4:**
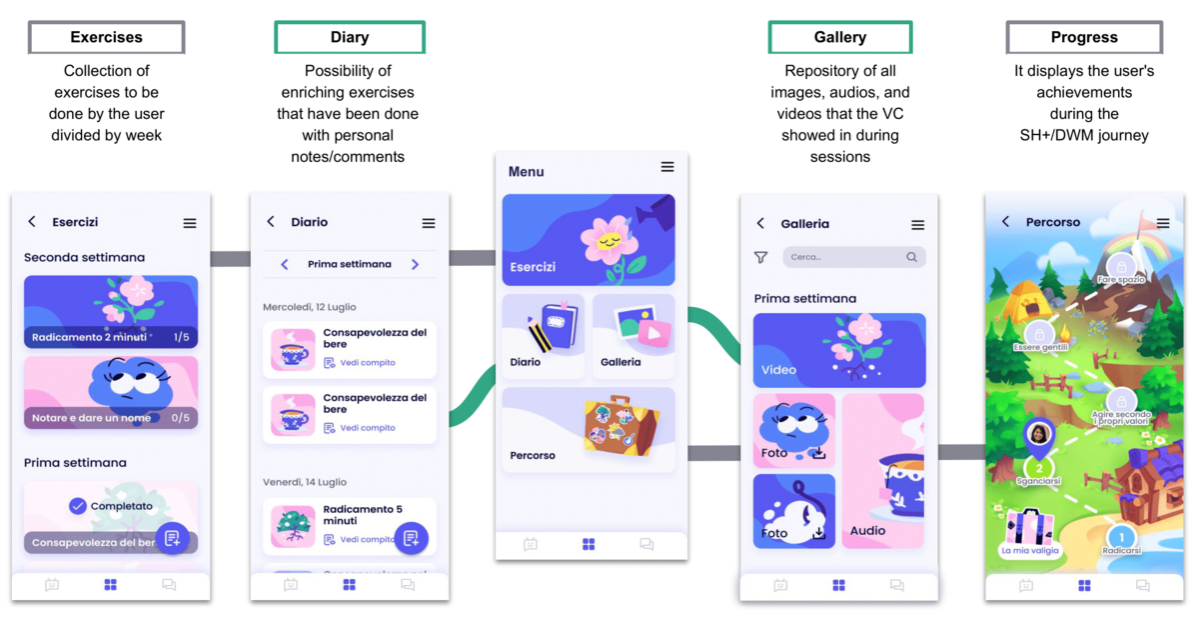
Automated Well-Being Assistant (ALBA) app mock-ups of different sections (Exercises, Diary, Principal Menu, Galley, and Progress). DWM: Doing What Matters in times of stress; SH+: Self-Help Plus; VC: virtual coach.

The preliminary outcomes derived from the initial adaptation phase show a promising development trajectory. Our previous co-design study validated the chatbot’s dialogues for effective content delivery [44]. Positive feedback highlighted the app’s structure, user-friendly design, and perceived credibility. Key innovations included interactive elements, gamification, and personalized feedback. Methodical selections for facilitating the transition from manual modality to the application domain are shown in prototypical mock-up representations in Figure 4. The app is poised to feature a subdued color palette, coupled with cartoonish character renditions, strategically created to alleviate the emotional burden of embarking on such a path to manage stress [50]. Moreover, this stylish choice also aligns with the cartoonish characters of the DWM booklet.

The app is poised to be compartmentalized into discrete segments, the utility of which will undergo rigorous evaluative scrutiny in a methodologically standardized manner, as delineated in the co-design investigations pertaining to Phase Ib of the ORBIT model. Subsequently, a comprehensive evaluation of usability and acceptability will be undertaken during Phase II. These future phases will consist of a plan encompassing the scrutiny of the app’s prototypes through successive stages of deployment. This sequential study will initially involve a limited cohort of stakeholders, followed by an equivalently small sample of volunteer women contending with breast cancer and gestational conditions.

**Figure 5:**
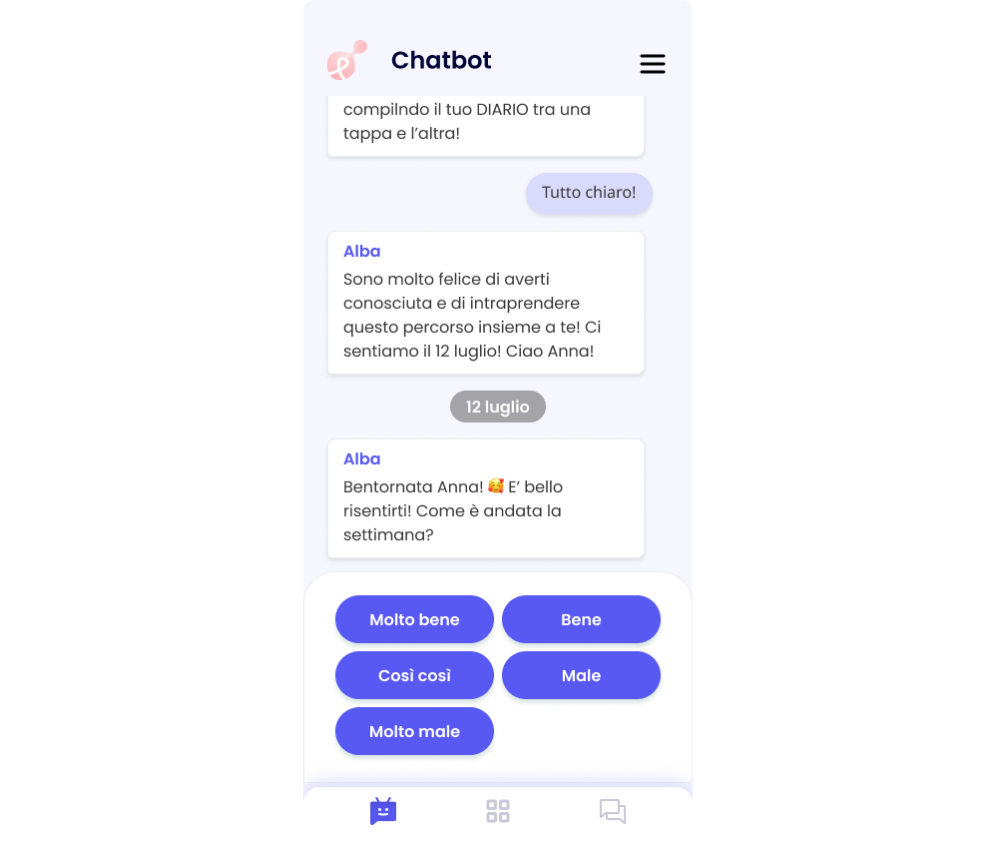
Automated Well-Being Assistant (ALBA)’s dialogue section with open-ended questions and button-based responses (guided answers).

The complete array of dialogues and transcripts constituting the ALBA virtual coach, originating from the SH+ manual and DWM booklet, has been fashioned using the methodologies expounded in the preceding sections. These textual compositions are presently undergoing the technical instantiation process. A selected mockup extracted from these dialogues is visually depicted in Figure 5, offering a glimpse into their compositional structure and stylistic attributes. Noteworthy features encompass a spectrum of question types, comprising both structured dialogue and open dialogue exchanges concurring to foster a heightened degree of interactivity and engagement within the communication exchange.

## Discussion

### Principal Findings

New technologies can improve the sustainability of the health care system by providing round-the-clock support to patients and optimizing the interventions provided by health care professionals [5]. Nevertheless, the innovations associated with developing and utilizing new technologies for mental health remain a subject of ongoing discussion. Empirical studies on evidence-based digital interventions for mental health, including internet-based CBT, have shown that these interventions are effective, feasible, and acceptable to users [51-53]. However, some limitations have been found, mainly regarding users’ low engagement and completion rates [51,54]. Integrating human coaching and support in digital mental health interventions can help improve adherence and behavior change outcomes [55,56], although this may reduce the scalability of such solutions. In this optic, the use of chatbots for digital mental health interventions has attracted interest in the design community. A growing number of studies are reporting their acceptability and feasibility for users [57,58], as well as their effectiveness in reducing perceived stress [54], thereby improving symptoms of anxiety [59-61], depression [60,61], and insomnia [62]. A recent meta-analysis suggested that internet-delivered CBT is likely as effective as conventional face-to-face CBT. However, there are still unanswered questions about treatment moderators and implementation strategies [63]. Another study highlights the importance of understanding and accommodating diverse patient preferences when implementing self-management applications in cancer care. It underscores the significance of patient engagement in self-help and suggests that digital interventions can empower individuals to control their well-being. However, participant experiences varied, with differing perceptions of added value. Recommendations include refinements for improved user-friendliness and tailoring content to individual preferences through continuous evaluation and collaboration with end users to enhance intervention effectiveness and user experience [64].

In line with these new emerging technological tools to face sociopsychological demands, this paper seeks to provide insights beyond the end user interfaces, delving into the complexities of an mHealth app for stress management development, ALBA. This ongoing research adapts the transdiagnostic SH+ package to a chatbot intervention for women with breast cancer and pregnant women in Italy. ALBA replaces human facilitators, delivering text, audio, video, and graphics content; guiding participants; initiating interactions; and ensuring exercise implementation. The adaptation process involved careful consideration of cultural nuances and the psychological states of the target populations. The adaptation process required evaluating psychological states, tailoring materials, and customizing content for the users’ specific needs. Moreover, the intervention was adapted to individual support: The chatbot-mediated approach aims to provide a resource-efficient and innovative way to help through digital translation, allowing flexible session delivery with personalized sessions and reminders to enhance adherence to the stress management path. The chatbot incorporates gamification elements, assigning homework, monitoring progress, and providing feedback through a diary section with badges as favorable reinforcement. Gamification principles enhance engagement and motivation, making the intervention more effective and enjoyable for end users.

This research underscores the inherent value of consistent daily engagement with virtual coaches, surpassing sporadic interactions with human specialists and thus mitigating constraints stemming from their limited availability [2,3]. Moreover, considering the link between costs and human interaction, the shift from group-based interventions to individualized approaches is explored. Although this shift may increase accessibility and affordability, it simultaneously triggers reflections on the communal synergy and interpersonal involvement integral to group interventions. Nevertheless, the chatbot offers a prospect to decipher the precise impact of virtual coaching interventions not burdened by an array of confounding variables [16]. This study also contributes to understanding their potential within the mental health landscape by delineating the nuanced advantages and considerations surrounding virtual coaching applications.

Another aim is to draw clinical attention to new target populations’ stress triggers and management through the delicate and underestimated design phases. In all the adaptation steps, the end user viewpoint was considered. The research emphasizes the importance of considering end users and their perspectives in enhancing the accessibility of the intervention through a user-friendly, chatbot-guided system. The commitment to tailoring the intervention for Italian women is a comprehensive approach aimed at meeting the specific needs of the target demographic. Indeed, the need for support from these two populations is increasingly evident. The transposition of CBT techniques into digital tools is the most functional according to the literature both for pregnant women [65] and women with a breast cancer diagnosis [35], decreasing anxiety and depression symptoms. The application of digital CBT therapies is still open to experimentation, and this digital intervention will be included in this panorama.

### Challenges and Limitations

The adaptation work for a digital stress management app has highlighted several practical challenges. One significant challenge is the need for intermediaries to guide during or between sessions, necessitating the anticipation and embedding of potential user queries or uncertainties within the application framework. To address difficulties such as comprehension issues, the app will offer feedback based on a manual and provide avenues for further assistance, including technical support or access to a psychologist.

Engagement maintenance during the transition from group to individual digital sessions is another key challenge addressed through gamification elements and interactive exercises with feedback and reminders. Subsequent empirical investigations are needed to validate these strategies and assumptions.

Future research questions and challenges include participant recruitment and privacy concerns. Although stress management’s universal relevance aids recruitment prospects, obstacles may arise from stigma and health care access barriers. Alternative recruitment channels such as voluntary participation and partnerships are being explored.

### Further Developments

ALBA will adhere to the successive stages of the ORBIT model. The initial emphasis will be on Phase Ib, followed by the comprehensive redefinition of components through standardized co-design investigations. Subsequent efforts will encompass the methodical assessment of prototype usability, in terms of app architecture, design iterations, and technical performance, and the evaluation of app efficacy after its revision in randomized control trials. These steps are needed to understand the user experience and acceptability of the app, proving its clinical efficacy and facilitating its possible successful implementation in real-world settings with these specific target populations. Every specification inherent to possible end user app utilization will be deeply considered and empirically validated.

An additional advancement involves examining the practical implications of utilizing this app within real-world settings. This would encompass a comprehensive evaluation of the intervention’s efficacy and efficiency in rendering support and facilitating progressive care for the designated populations. It could shed light on sustainability in terms of the resource allocation and human capital expenditures stemming from implementing this preventative and low-intensity support framework. From a broader perspective, the potential exists for the creation of analogous applications, exhibiting tailored cultural and linguistic adaptations to address the needs of diverse target demographics, encompassing women who have breast cancer as well as pregnant women from varying cultural backgrounds.

### Conclusions

Introducing ALBA, a new mobile tool designed for some vulnerable female populations, enriches the mHealth scientific literature. This innovation involves implementing an SH+ validated intervention using a new delivery tool, incorporating the previously outlined and targeted modifications. Using the SH+ protocol will shed light on the exploration in assessing the efficacy of the chatbot as an innovative intervention strategy, along with evaluating the impact of the evidence-based intervention on these specific groups. The commitment extends to comprehensive implementation research, delving into clinical outcomes and examining app implementation-related factors such as feasibility, fidelity, and accessibility.

## Appendix

Multimedia Appendix 1 Description of the adaptation from SH+ to ALBA.

## Abbreviations

ALBA: Automated Well-Being Assistant
CBT: cognitive behavior therapy
DTx: digital therapeutic
DWM: Doing What Matters in times of stress
mHealth: mobile health
ORBIT: Obesity-Related Behavioral Intervention Trials
SH+: Self-Help Plus
WHO: World Health Organization

## Data Availability

The data generated and analyzed during the study are available from the corresponding author upon reasonable request. Before disclosure, data are aggregated and anonymized.
