# Supplementary material for: World Health Organization Evidence-Based Self-Help Plus Intervention for Stress Management via Chatbot: Protocol for Adaptation to a Tech-Enabled Model
Source: JMIR Res Protoc. 2025 Jun 26;14:e69644. doi: 10.2196/69644 (PMC12246761; doi:10.2196/69644)
Supplement: Multimedia Appendix 1 [file resprot_v14i1e69644_app1.docx]

**Table S1.** Description of the adaptation from SH+ to ALBA. The table’s columns, starting from the conceptual Elements that make up the SH+ protocol, elucidate the modifications made in transitioning from the SH+ intervention to ALBA application. This is achieved by describing the overall Digital translation process and the significance of this adaptation step, referred to as Porting.

|  | **Elements** | **SH+ →** | **→ Digital translation →** | **→ Porting →** | **→ ALBA** |
| --- | --- | --- | --- | --- | --- |
| **Adaptation from SH+** | **Content** | Manuals (SH+ and DWM) with figures and audios,  pre-recorded sessions’ materials | Mobile app with dialogues and multimedial resources | Sustainable and accessible DTx intervention [1-7] | Original contents are displayed inside the app, dialogues of sessions, visual and audio multimedia resources are accessible in different section of the application (such as Gallery or Exercises’ sections) |
|  | **Guide** | Human guides: Two facilitators  in-person (SH+ protocol); Helpers’ phone calls (RESPOND project) | Chatbot guided intervention | No need for human resources, both for training of facilitators/helpers and the following guided sessions and supervision [5,6]  Intuitiveness and naturalness of chatbot dialogue-based interaction [8,9] | The ALBA chatbot leading interventions modules |
| **Adaptation and needs of the target populations** | **Participant** | Participant: person experiencing stressful situation (e.g., refugees), group member | Italian single participant experiencing stressful situation and app end-user | Individualization of the intervention with more attention to personalization  No desiderability or external pressures effects [10] | End user: breast cancer patient or pregnant woman, individual intervention with female adapted content |
|  | **Session** | In presence 2-hours sessions on established weekly appointment | Chatbot dialogues to release sessions | Self-help at home, intervention in the end-user hands  Possibility of personalization of sessions’ day for dialogues delivery [5,10,11] | At home interactive dialogues with ALBA  Dialogues duration of 40 minutes (possibility to decide the day of weekly interaction and split them in two subsequent days) |
| **Key Points from chatbot and m-health literature** | **Homework** | Participant are requested to do individual homework (no monitored in SH+; phone calls’ monitoring in RESPOND) | Requested homework with monitoring reminders, feedback and a diary section. | The end user is monitored and assisted in doing her homework to give strength and continuity to the self-help intervention [12,13] | Check and notifications for exercises to do between dialogues' sessions, possibility to do some homework with ALBA assistance and additional motivational dialogues to help the user, more support in initial phases |
|  | **Engagement** | Being part of a group and face to face intervention, strong inner motivation to participate and reach the sessions’ location | Interactivity and gamification aspects of the chatbot dialogues and intersession monitoring, inner motivation to participate | The interest in the intervention is kept alive during the dialogues with interactive answers modalities and during the intersession week through an adequate level of assistance and monitoring. Gamification aspects extrinsically encourage and motivate the user to going on in the intervention [12-14] | ALBA dialogues interactivity through user’s possibility to answer in different ways (buttons, open and closed answers)  ALBA app gamification: badges as prizes, advance bar as journey metaphor.  Monitoring and motivational feedback and dialogues |

**References**

1. Zhang X, Li Y, Wang J, Mao F, Wu L, Huang Y, Sun J, Cao F. Effectiveness of digital guided self-help mindfulness training during pregnancy on maternal psychological distress and infant neuropsychological development: randomized controlled trial. J Med Internet Res 2023 Feb 10; 25:e41298
2. Torous J, Chan SR, Yee-Marie Tan Shih, Behrens J, Mathew I, Conrad EJ, Hinton L, Yellowlees P, Keshavan M. Patient smartphone ownership and interest in mobile apps to monitor symptoms of mental health conditions: a survey in four geographically distinct psychiatric clinics. JMIR Ment Health 2014; 1(1):e5
3. Davies EB, Morriss R, Glazebrook C. Computer-delivered and web-based interventions to improve depression, anxiety, and psychological well-being of university students: a systematic review and meta-analysis. J Med Internet Res 2014 May 16; 16(5):e130
4. Zhang MW, Ho RC. Moodle: the cost effective solution for internet cognitive behavioral therapy (I-CBT) interventions. THC 2017 Feb 21; 25(1):163-165
5. Baños RM, Herrero R, Vara MD. What is the current and future status of digital mental health interventions?. Span. J. Psychol 2022 Feb 02; 25:1
6. Taylor CB, Fitzsimmons-Craft Ellen E, Graham AK. Digital technology can revolutionize mental health services delivery: the COVID-19 crisis as a catalyst for change. Int J Eat Disord 2020 Jul 25; 53(7):1155-1157
7. Barrera AZ, Wickham RE, Muñoz Ricardo F. Online prevention of postpartum depression for Spanish- and English-speaking pregnant women: a pilot randomized controlled trial. Internet Interv 2015 Sept 01; 2(3):257-265
8. Suganuma S, Sakamoto D, Shimoyama H. An embodied conversational agent for unguided internet-based cognitive behavior therapy in preventative mental health: feasibility and acceptability pilot trial. JMIR Ment Health 2018 Jul 31; 5(3):e10454
9. Vaidyam AN, Wisniewski H, Halamka JD, Kashavan MS, Torous JB. Chatbots and conversational agents in mental health: a review of the psychiatric landscape. Can J Psychiatry 2019 Jul; 64(7):456-464
10. Recchia G, Maria Capuano D, Mistri N, Verna R. Digital therapeutics-what they are, what they will be. Act Scie Medic 2020 Feb 26; 4(3):01-09
11. Bucci S, Schwannauer M, Berry N. The digital revolution and its impact on mental health care. Psychol Psychother 2019 Jun 28; 92(2):277-297
12. van Dulmen Sandra, Sluijs E, van Dijk Liset, de Ridder Denise, Heerdink R, Bensing J. Patient adherence to medical treatment: a review of reviews. BMC Health Serv Res 2007 Apr 17; 7:55
13. Titov N, Dear BF, Johnston L, Lorian C, Zou J, Wootton B, Spence J, McEvoy PM, Rapee RM. Improving adherence and clinical outcomes in self-guided internet treatment for anxiety and depression: randomised controlled trial. PLoS One 2013; 8(7):e62873
14. De Croon R, Geuens J, Verbert K, Abeele VV. A Systematic Review of the Effect of Gamification on Adherence Across Disciplines. 2021. Presented at: HCI in Games: Experience Design and Game Mechanics: Third International Conference; July 24-29, 2021; Virtual event.
